# Immune Thrombocytopenic Purpura Associated With Systemic Lupus Erythematosus, Helicobacter pylori, and Hepatitis B

**DOI:** 10.7759/cureus.56411

**Published:** 2024-03-18

**Authors:** Bamidele O Johnson, Amisha Nimawat, Nyier W Doar, Thi Nguyen, Malar Thwin

**Affiliations:** 1 Psychiatry and Behavioral Sciences, Interfaith Medical Center, Brooklyn, USA; 2 Internal Medicine, Interfaith Medical Center, Brooklyn, USA

**Keywords:** h. pylori infection, systemic lupus erythematosus, anti-ds dna, intravenous immunoglobulins (ivig), triple therapy, triple therapy for h. pylori, adamts 13, hepatitis b infection, thrombopoietin receptor agonist (tpo-ra), immune mediated thrombocytopenic purpura (itp)

## Abstract

Immune thrombocytopenic purpura (ITP) is a hematologic condition characterized by decreased circulating platelets, resulting in bruising, bleeding gums, and internal bleeding. This disorder can be categorized into two primary forms based on the duration of symptoms and underlying causes. Acute ITP primarily affects young children, typically between the ages of two and six, but it can also impact older children and adults. Viral infections like chickenpox, respiratory infections, or gastroenteritis often precede it. Acute ITP manifests suddenly and lasts for a short period, typically less than six months and sometimes only a few weeks.

On the other hand, chronic ITP primarily affects adults but can occur at any age, including childhood and adolescence. The main characteristic of chronic ITP is the persistence of symptoms for more than six months. It can be either idiopathic (primary), with no discernible etiologic cause, or secondary to various conditions such as autoimmune diseases (e.g., systemic lupus erythematosus), viral infections (e.g., human immunodeficiency virus (HIV), hepatitis C virus (HCV)), certain malignancies (e.g., chronic lymphocytic leukemia), or drug reactions. This case report presents the management of a 36-year-old African American female diagnosed with ITP associated with systemic lupus erythematosus, Helicobacter (H.) pylori, and hepatitis B infection.

## Introduction

Immune thrombocytopenia (ITP) purpura, formerly known as idiopathic thrombocytopenia purpura, is defined according to the American Society of Hematology as an autoimmune disorder that is characterized by isolated thrombocytopenia, either due to platelet destruction or impaired production in the setting of a generalized purpuric rash with normal WBC count and normal hemoglobin [1].

The classification of ITP can be based on the duration of symptoms and underlying causes [2]. Primary ITP, which accounts for approximately 80% of cases, occurs without an identifiable cause or underlying disorder [1,3]. The development of primary ITP is theorized to involve the loss of self-tolerance, leading to an autoimmune process affecting innate and adaptive immune responses [3,4]. In contrast, around 20% of ITP cases are classified as secondary, meaning they occur in association with an underlying cause or condition [4]. Secondary ITP can be triggered by factors such as certain medications (e.g., carbamazepine, phenytoin, rifampin), malignancies (e.g., adenocarcinoma and lymphoma), autoimmune diseases (e.g., systemic lupus erythematosus, autoimmune hepatitis, thyroid disease), or infections (e.g., human immunodeficiency virus (HIV), hepatitis) [1,4]. In these cases, the formation of anti-platelet antibodies leads to the destruction of platelets [1,2,4].

The International Working Group on ITP has established clinical phases to define the different stages of the disease [5]. Newly diagnosed or acute ITP refers to the initial three months after diagnosis, persistent ITP lasts 3 to 12 months, chronic ITP extends beyond 12 months, and refractory ITP denotes splenectomy failure as a treatment option [1,3].

The acute form primarily affects children, with equal prevalence in both sexes and is often preceded by a viral infection [1,5]. Antibodies produced against viral or bacterial antigens can cross-react with normal platelet antigens, resulting in molecular mimicry [2,4]. Common viral infections associated with acute ITP include HIV, hepatitis C, cytomegalovirus, and varicella zoster [1,2,4]. Most children with acute ITP experience a benign course and spontaneous recovery within three months, typically requiring no treatment [1,3,5].

On the other hand, chronic ITP predominantly affects adolescents and young adults (18 to 45 years old), with a higher incidence in females as compared to males (ratio of 3 to 1) [2,4]. The greater prevalence of autoimmune diseases in women may contribute to this female preponderance [4].

The annual incidence of ITP among adults is estimated to be between 1 and 6 cases per 100,000 individuals while the prevalence is approximately 12 cases per 100,000 individuals [1]. Incidence rates peak around age 60, but the likelihood of developing ITP increases with age [3]. After 60 years, the incidence is similar for males and females [1,3].

The etiology of ITP is believed to involve autoantibodies, primarily immunoglobulin G (IgG), which target platelet membrane proteins such as glycoprotein (GP) IIb/IIIa complex, GP Ib/IIa, and GP VI [1,6]. These antibodies destroy platelets as they are cleared by tissue macrophages in the spleen, leading to a shortened platelet lifespan and subsequent thrombocytopenia [4,6]. Another proposed mechanism involves T-cell-mediated cytotoxicity, where cytotoxic T-cells attack megakaryocytes in the bone marrow [1,4,6]. ITP is associated with preexisting autoimmune conditions like systemic lupus erythematosus, antiphospholipid syndrome, and common variable immunodeficiency [4-7].

Systemic lupus erythematosus (SLE) and immune thrombocytopenia (ITP) are two distinct medical conditions that can affect individuals, particularly women, in different ways [7]. Approximately one-third of SLE patients experience thrombocytopenia, a condition characterized by a low platelet count, during their illness [7,8]. Conversely, SLE develops in around 2% to 5% of ITP patients monitored over an extended period [8,9]. The development of thrombocytopenia in SLE patients is attributed to various factors, including the presence of antibodies targeting platelet glycoproteins, DNA, phospholipids, phospholipid-binding proteins, CD40 ligand, thrombopoietin, and its receptor, as well as immune complexes with diverse compositions [7-9]. Impaired apoptosis mediated by the FAS-FAS ligand pathway also contributes to this condition [7,8].

Helicobacter pylori (HP), a bacterium associated with gastrointestinal infections, has been strongly linked to the development of ITP [10]. The pathogenesis of ITP-HP is theorized to involve molecular mimicry, where HP produces antibodies that cross-react with platelets [4]. Geographic variation in the virulence genes of Helicobacter pylori (HP), specifically the vacuolating cytotoxin gene A (VacA) and cytotoxin-associated gene A (cagA) has been observed [11]. This variation is associated with the incidence of immune thrombocytopenia associated with H. pylori infection (ITP-HP) [10,11]. The prevalence of HP infection among ITP patients is similar to that of the general population when considering age and location [1,6]. However, only a tiny fraction of infected individuals develop ITP-HP [11]. Platelet response to documented bacterial eradication varies across studies, ranging from 0% to 7% in the US series and up to 100% in the Japanese series [10-12]. Successful bacterial eradication has been shown to provide benefits compared to unsuccessful or no eradication [12,13]. Failure to correct thrombocytopenia in some cases is associated with the emergence of antigen-independent autoreactive clones of T and B cells [11,13]. HP infection may also affect the balance of activating and inhibiting FcγRs (Fc gamma receptors), and platelet levels often increase within a few weeks of antibiotic treatment, potentially due to the restoration of this balance [11-13].

In addition, viral hepatitis infections, specifically hepatitis B and C, have been associated with immune thrombocytopenia (ITP) through various mechanisms, including molecular mimicry, epitope spreading, and immune dysregulation [14]. Interestingly, patients with hepatitis C virus (HCV) or hepatitis B virus (HBV) have a higher likelihood of being infected with Helicobacter pylori compared to individuals with autoimmune liver cirrhosis (ALC) or primary biliary cirrhosis [14,15]. A study by Salehi et al. (2014) revealed that patients who underwent H. pylori eradication regimens experienced reduced liver enzyme levels. This suggests a potential link between H. pylori infection and liver function in individuals with viral hepatitis [12].

## Case presentation

**Figure 1:**
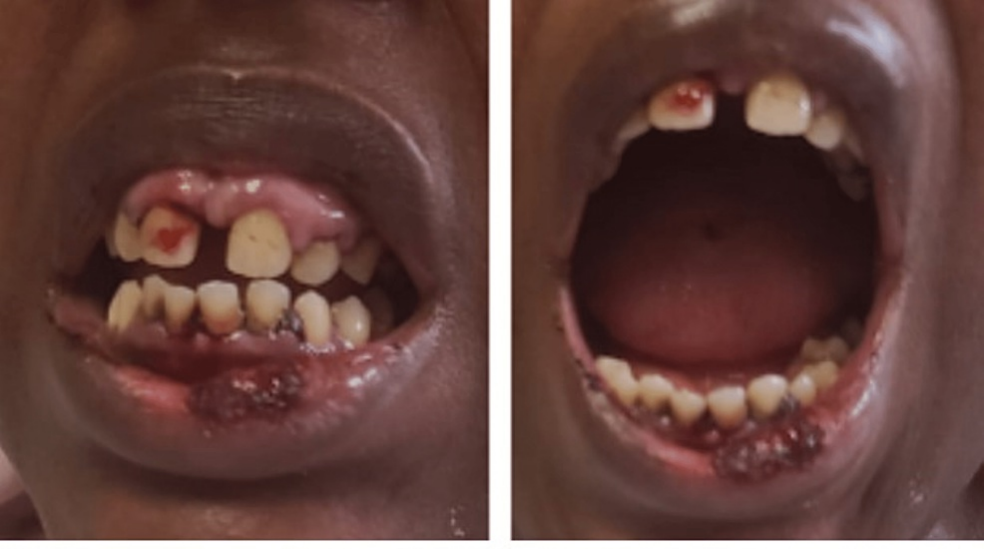
Mucocutaneous bleeding and gum hypertrophy

A 36-year-old female with a medical history of hypertension, asthma, and ovarian cysts, was referred by her primary care physician for further assessment of thrombocytopenia and the need for platelet transfusion. The patient was diagnosed with thrombocytopenia approximately two months prior, with an initial platelet count of 7000/µl. She presented with recurrent bleeding episodes from the mouth and nose, heavy menstruation accompanied by blood clots, lightheadedness, dizziness, joint pain, unintentional weight loss, decreased appetite, and malar rash. Physical examination revealed gum hypertrophy and bleeding gums, as shown in Figure 1. Other findings included bruises on the lower limbs and tender cervical lymph nodes. 

Diagnostic workup

Laboratory tests were performed upon presentation, indicating a platelet count of 4000/µl. A comprehensive metabolic panel and a complete blood count panel were ordered and monitored throughout the patient’s hospital stay, as shown in Table 1. Additional investigations were ordered to determine the underlying cause of thrombocytopenia, including screening for a hepatitis panel, H. pylori stool antigen test, HIV, antinuclear antibody (ANA) comprehensive panel, anti-platelet antibodies, beta-2 microglobulin, and coagulation studies, as shown in Table 2. A panoramic CT scan was also conducted.

**Table 1:** Trends in complete blood count and complete metabolic panel

	Normal value	Hospital Day 1	Hospital Day 4	Hospital Day 11	Hospital Day 13	Hospital Day 14	Hospital Day 15
Sodium (Na)	135-137 mmol/L	136	135	137	133 (L)	135	135
Potassium (K)	3.5-5.3 mmol/L	3.4 (L)	3.7	4.8	4.5	4.0	3.1 (L)
Chloride (Cl)	96-109 mmol/L	100	105	105	103	101	102
Calcium (Ca)	8.6-10.2 mg/dL	9.2	8.5 (L)	9.2	8.8	9.0	8.7
Anion gap (AG)	8-16 mEq/L	10	4	10	7.00	8.00	8.00
Glucose	70-110 mg/dL	95	108	113 (H)	86	91	107
Blood urea nitrogen (BUN)	7-20 mg/dL	12	19	22 (H)	17	16	13
Creatinine (Cr)	0.7-1.2 mg/dL	0.9	0.7	0.7	0.6	0.64	0.7
Aspartate aminotransferase (AST)	<40 U/L	45 (H)	18	26	33	33	28
Alanine aminotransferase (ALT)	<45 U/L	24	13	30	35	42	44
Alkaline phosphatase (ALP)	20-140 U/L	61	40	85	70	101	65
Bilirubin, total	<1.2-1.3 mg/dL	0.8	0.4	0.4	0.5	0.5	0.3
Estimated glomerular filtration rate (eGFR)	>90	85 (L)	114.9	>90	>90	>90	114.9
Protein, total	6-8.5 g/dL	8.3	8.5	9.9 (H)	8.6 (H)	8.5	7.3
White blood cell count (WBC)	4.5-10/mcL	3.8 (L)	5.2	10.9 (H)	12.4 (H)	17.9 (H)	20.1 (H)
Red blood cell count (RBC)	4.2-5.4/mcL	3.77 (L)	3.17 (L)	3.57 (L)	3.94 (L)	3.77 (L)	3.55 (L)
Hemoglobin (Hgb)	12.1-15.1 g/dL	11.3	9.5 (L)	10.9 (L)	11.9	11.3 (L)	10.4 (L)
Hematocrit (Hct)	36.1-44.3%	34.4 (L)	28.9 (L)	33.4 (L)	36.8 (L)	35.0 (L)	32.6 (L)
Mean corpuscular volume (MCV)	80-95 fl	91.3	91.1	93.5	93.6	93.0	91.8
Mean corpuscular hemoglobin (MCH)	27-31 pg/cell	29.8	30.0	30.6	30.4	30.1	29.3
Mean corpuscular hemoglobin concentration (MCHC)	32-36 g/dL	32.7	32.9	32.7	32.4	32.4	31.9
Red cell distribution width (RDW)	12-15%	14.7	14.7	15.3 (H)	15.6 (H)	15.4 (H)	15.2 (H)
Mean platelet volume (MPV)	7.5-11.5 fl	13.1 (H)	11	11.9 (H)	14.8 (H)	13.6 (H)	10.7
Platelet (PLT)	150-450/mcL	4 (LL)	5 (LL)	6 (LL)	41	70	155

**Table 2:** Other laboratory investigations during admission

	Patient’s value	Normal value
Prothrombin time (PT)	11.9 seconds	11-13.5 seconds
Internationalized normal ratio (INR)	1.00	<1.1
Partial thromboplastin time (PTT)	32.4 seconds	25-35 seconds
Erythrocyte sedimentation rate (ESR)	41 mm/hr (H)	1-20 mm/hr
Reticulocyte count %	2.68 % (H)	0.5-2%
Reticulocyte count absolute	0.1016 (H)	0.024-0.09
Immature reticulocyte fraction (IRF)	0.65	2.3%-13.4%
Anti-double stranded DNA	12 IU/ml (H)	<10 IU/ml
Anti-cardiolipin IgM	13 U/ml (H)	0-12 U/ml
Antibody- U1-ribonucleoprotein (U1 RNP)	1.3 AI (H)	<1 AI
Anti-smith antibody	0.2 AI	<1 AI
Anti scleroderma-70	<0.2 AI	<1 AI
Sjogren’s anti-SS-A	>8.0 AI (H)	<7 AI
Sjogren’s anti-SS-B	<0.2 AI	<7 AI
Anti-centromere B	<0.2 AI	<1 AI
Anti-chromatin	0.8 AI	<1 AI
Anti-JO-1	<0.2 AI	<1 AI
Hepatitis B core total Ab	Positive	
Hepatitis A total Ab	Positive	
Hepatitis B surface antigen	Non-reactive	
Hepatitis B surface antibodies	Positive (>500)	
Beta-2 microglobulin	4.2 mg/L (H)	0.6-2.4 mg/L
Mycoplasma pneumoniae IgG antibodies	469 U/ml (H)	0-99 U/ml
Glycoprotein (GP IIb/IIIa)	Negative	
Glycoprotein (GP Ia/IIa)	Negative	
Glycoprotein (GP Ib/IX)	Negative	
Human lymphocyte antigen (HLA class I)	Negative	
Glycoprotein P (GP IV)	Negative	
Rheumatoid factor (RF)	Non-reactive	
Complement C3, serum	103 mg/dL	80-160 mg/dL
Complement C4, serum	11 mg/dL (L)	12-72 mg/dL
Anti-CCP Ab, IgG + IgA	<20 U	<20 U
Anti-histone	2.4 U (H)	0-0.9 U
Anti-parietal cell antibody	43.5 U (H)	0-20 U/ml
AdamTS 13 antibody	12 U/ml (H)	<12 U/ml
AdamTS 13 activity	80.1 %	>70%
Direct Coombs	Negative	

Treatment and management

The patient received Intravenous methylprednisolone 10 mg, and 1 unit of platelets was transfused to achieve a target platelet count above 10,000/µl. The patient's platelet count increased to approximately 6000/µl on the second day, necessitating another platelet transfusion. Intravenous methylprednisolone 10 mg was changed to intravenous Decadron 40 mg daily to be received for four days, and intravenous immunoglobulin (IVIG) 400 mg/kg daily was scheduled for five days. As shown in Table 2, the patient’s ANA panel revealed elevated anti-ds DNA antibodies and anti-SS-A antibodies, positive hepatitis B core IgM antibodies, elevated beta-2 microglobulin levels, and positive H. pylori antigen test. The anti-platelet antibodies assay was negative. Additional tests were ordered to aid the diagnosis, including a peripheral smear, flow cytometry, and bone marrow biopsy.

**Figure 2:**
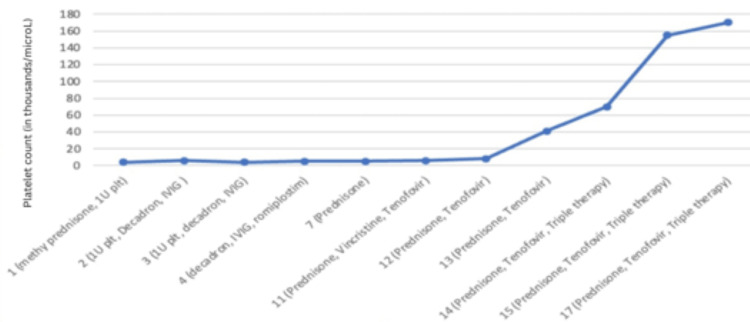
Platelet count (in thousands/microL) vs. hospital days/treatment

On the third day, the patient received another platelet transfusion in preparation for the bone marrow biopsy. HBV PCR was requested to assess the patient's hepatitis B status. Parietal cell antibodies were positive while intrinsic factor antibodies were negative. The CT findings revealed fatty liver, mild splenomegaly, and lymphadenopathy in bilateral axilla and hilar regions. The patient's platelet count increased to 8000/µl, as shown in Figure 2. The rheumatology team was consulted to investigate the possibility of systemic lupus erythematosus, ordering a rheumatology panel consisting of complement levels (C3 and C4), anti-histone antibodies, rheumatoid factor, and anti-CCP antibodies.

On the fourth day, the patient commenced treatment with thrombopoietin receptor agonists (TPO-RAs), romiplostim 120mcg subcutaneously weekly for four weeks. The patient continued to receive the same doses of intravenous decadron and IVIG. HBV PCR results returned negative. Peripheral smear analysis revealed no blasts, platelets, clumps, or fragmented RBCs. Flow cytometry ruled out leukemia/lymphoma. The patient's platelet count remained at 5000/µl, as shown in Figure 2. 

On the seventh day, intravenous decadron was switched to oral prednisone 80 mg daily, with a plan to taper the dosage gradually. Due to the low platelet count, the hematology team suggested plasmapheresis, platelet-stimulating, and immunomodulatory agents like rituximab. Further laboratory findings indicated low C4 levels, elevated anti-histone antibodies, and a borderline high ADAM TS13 antibody, as shown in Table 2. However, ADAM TS13 activity fell within the normal range.

On the eleventh day, the patient's platelet count remained low at 7000/µl, as shown in Figure 2 below. Bone marrow biopsy reports did not reveal significant findings. Considering the patient's hepatitis panel results, consultation with the gastroenterology department confirmed previous exposure to hepatitis B without current active infection. Therefore, to prevent viral reactivation, the patient was started on oral tenofovir 300 mg daily for two weeks before starting rituximab treatment. As an immunosuppressive agent, vincristine 2 mg intravenous push was administered, with a plan for two more cycles over three weeks.

On the twelfth day, the patient's platelet count was 8000/µl, as shown in Figure 2 below. Oral prednisone 80 mg daily and oral tenofovir was continued. Due to the sustained thrombocytopenia, the surgical team was consulted to evaluate the potential need for splenectomy in case of treatment failure.

The platelet count significantly improved to 41,000/µl on the thirteenth day, as shown in Figure 2 below. However, the H. pylori antigen test yielded a positive result. Therefore, on the fourteenth day, the patient was started on triple therapy consisting of oral metronidazole 500 mg every 8 hours, clarithromycin 500 mg every 12 hours, and pantoprazole 40 mg twice daily for 14 days to eradicate the H. pylori infection. The platelet counts increased to 70,000/µl on the fourteenth day, as shown in Figure 2.

On the fifteenth day, the patient continued prednisone 80 mg, oral tenofovir 300 mg, and triple therapy, and the platelet count reached 155,000/µl, as shown in Figure 2.

On the seventeenth day, the platelet count improved to 170,000/µl, and the patient was discharged with an outpatient follow-up plan. The graph depicted in Figure 2 shows the platelet count trend against the treatment the patient received on respective days in the hospital.

## Discussion

Thrombocytopenia can be classified based on the pathophysiological mechanisms involved such as decreased platelet production, increased platelet destruction, or sequestration of platelets [1,4,5]. In this case, the patient presented with severe thrombocytopenia with bleeding manifestations, necessitating prompt evaluation and management. The initial platelet count of 7000/µl and subsequent drop to 4000/µl raised concerns for ongoing platelet destruction or impaired production.

The patient's history of hypertension, asthma, and ovarian cysts did not provide a clear etiological link to thrombocytopenia. Diagnosing the cause of thrombocytopenia can be difficult [8]. When there is no evidence of an underlying pathology, primary immune thrombocytopenia is often considered the diagnosis [1,3]. Therefore, a comprehensive diagnostic workup was performed to identify the underlying cause. The initial laboratory tests included screening for common viral infections, autoimmune markers, and platelet antibodies. The patient received platelet transfusions to maintain a safe platelet count while awaiting further investigations.

The subsequent findings revealed several significant abnormalities. The presence of elevated anti-ds DNA antibodies and anti-SS-A antibodies in the ANA panel suggested an autoimmune component in the pathogenesis of thrombocytopenia. Additionally, the patient tested positive for hepatitis B core IgM antibodies, indicating a recent exposure or acute infection. These results prompted consultation with the gastroenterology department to evaluate the hepatitis panel further.

Further investigations, including peripheral smear analysis, flow cytometry, and bone marrow biopsy, were performed to assess for hematologic malignancies and other underlying causes. However, these tests did not reveal any significant abnormalities. The patient's platelet count remained low despite the various interventions, necessitating the consideration of alternative treatment strategies.

Identifying low C4 levels, elevated anti-histone antibodies, and a borderline high ADAM TS13 antibody raised the suspicion of an immune-mediated process contributing to thrombocytopenia. However, the ADAM TS13 activity within the normal range made diagnosing thrombotic thrombocytopenic purpura less likely. Thrombocytopenia secondary to a microangiopathic process (thrombotic thrombocytopenic purpura or hemolytic uremic syndrome) was also excluded: absence of renal, neurological, gastrointestinal or other typical symptoms, lack of anemia, reticulocytosis or schistocytes, and otherwise normal organ functions. The hematology team decided to start plasmapheresis, platelet-stimulating agents, and immunosuppressive agents, considering the refractory nature of thrombocytopenia and the need to address the underlying immune dysregulation.

The commencement of rituximab therapy was deferred by the patient's previous exposure to hepatitis B. The patient was treated with an antiviral agent, tenofovir therapy, before considering rituximab treatment in order to prevent viral reactivation. In the meantime, vincristine was administered as an alternative option, with plans for additional cycles if needed.

The positive H. pylori antigen test prompted the initiation of triple therapy consisting of metronidazole, clarithromycin, and pantoprazole. The patient's platelet count gradually improved with the continuation of oral prednisone and the initiation of eradication therapy for H. pylori infection. The subsequent increase in platelet counts demonstrated a positive response to the eradication therapy.

## Conclusions

This case report illustrates the rare but essential association between immune thrombocytopenia, systemic lupus erythematosus, and concurrent infections with Helicobacter pylori and hepatitis B virus. It highlights the challenges in diagnosing and managing immune thrombocytopenia in the setting of complex comorbidities, requiring a multidisciplinary approach. Prompt recognition and appropriate treatment of all contributing factors led to our patient's successful management and resolution of thrombocytopenia. Further research is warranted to understand better the interplay between immune-mediated disorders and infectious etiologies in thrombocytopenia.
